# Healthcare professionals’ knowledge, attitudes, and practice of podoconiosis management and associated factors in public hospitals in Ilu Ababor and Buno Bedelle zones, Southwest Ethiopia: a cross-sectional study

**DOI:** 10.3389/fpubh.2025.1454979

**Published:** 2025-02-24

**Authors:** Sanbato Tamiru, Yohanis Lulu, Kebebe Bidira, Bonsa Amsalu, Abdissa Duguma, Yared Nigusu, Wubishet Gezimu

**Affiliations:** ^1^Department of Nursing, College of Health Science, Mattu University, Mattu, Ethiopia; ^2^Armauer Hansen Research Institute, Addis Ababa, Ethiopia

**Keywords:** podoconiosis, management, healthcare professionals, Ilu Ababor, Southwest Ethiopia

## Abstract

**Background:**

Podoconiosis is non-filarial lymphoedema of the lower extremities. It impairs individuals’ overall lives, including their health, economy, psychology, and social interactions. Podoconiosis is a preventable and effectively manageable disease. The insights and skills of healthcare professionals are vital for the prevention and management of podoconiosis. However, healthcare professionals’ knowledge, attitudes, and practices of podoconiosis management have been poorly studied in Ethiopia.

**Objective:**

The study aimed to assess the healthcare professionals’ knowledge, attitudes, and practices toward podoconiosis management and associated factors in public hospitals in the Ilu Ababor and Buno Bedelle Zones.

**Methods:**

A facility-based cross-sectional study was conducted from December 1, 2022, to January 1, 2023. The data were collected from systematically selected health professionals using a self-administered structured questionnaire. Epi-data and SPSS were used for data entry and analysis, respectively. A binary logistic regression analysis was used to identify factors influencing the outcome variables. In the multivariable analysis, a *p*-value of ≤0.05 was used to pronounce statistical significance at a 95% confidence interval.

**Results:**

From the total of 399 participants involved, 262 (65.7%) had poor knowledge, 213 (54.3%) had unfavorable attitudes, and 308 (77.2%) had never practiced podoconiosis management. Years of experience, level of education, type of qualification, podoconiosis training, taking courses in education curricula, and attitudes were identified as factors influencing knowledge. The participants’ attitudes were influenced by age, podoconiosis training, receiving technical support, and knowledge. Their practices were significantly affected by age, education level, podoconiosis training, taking podoconiosis courses in educational curricula, and knowledge.

**Conclusion:**

The current study found poor healthcare professionals’ insights and skills in podoconiosis management in the study area. Therefore, concerned bodies need to set up capacity-building training and guidelines for healthcare professionals in the area to boost their knowledge, attitudes, and practice toward podoconiosis management.

## Introduction

Podoconiosis is a lymphedema on the lower extremities (foot) that affects a barefoot individual (1). It is the most common cause of tropical lymphedema following lymphatic filariasis (2). It is a non-infectious inflammatory response to the geochemical (smectite, mica, and quartz) substances found in red clay soil (1, 3). The lymphedema can occur on both extremities, however asymmetrical. It is characterized by firm nodules with itching, tingling, and flaring of the forefoot. Moreover, fibrosis, papillomatosis, and nodule-looking moss may be observed (4).

An estimated 4 million people are living with podoconiosis globally, mainly in Africa’s tropical countries, Central and South America, and Southeast Asia. Tropical African countries bear the highest disease burden. In Africa, the disease has been reported in Angola, Burundi, Cameroon, Cape Verde, Chad, the Democratic Republic of Congo, Equatorial Guinea, Ethiopia, Kenya, Madagascar, Mozambique, Niger, Nigeria, Rwanda, São Tomé and Príncipe, Sudan, Tanzania, and Uganda. Podoconiosis has been reported in the Latin American highlands in Brazil, Colombia, Costa Rica, Ecuador, El Salvador, French Guiana, Guatemala, Honduras, Mexico, Peru, and Suriname (5).

Of the 4 million global cases, about 1 million (a quarter of the global load of podoconiosis) are present in Ethiopia. Regionally, its prevalence is higher in the Ilu Ababor Zone (9.1%), Midakegni district of West Shewa Zone (7.4%), Pawe resettlement area (6%), Wolaita Zone (5.5%), Debreelias and Dembecha districts in the Amhara region (3.3%), and Gulliso district of West Wollega Zone (2.8%) (6). A community-based study conducted in the Bedelle district found 5.6% of podoconiosis (7). Geographically, the higher prevalence was observed in the central highlands of Ethiopia, including Amhara, Oromia, and the South Nation, Nationalities, and Peoples (SNNP) regional government. In addition, a higher prevalence was found in the south-western parts of the country (2).

Podoconiosis results in physical disability, psychological trauma, social stigma, and economic unproductivity (8). During acute inflammation, patients may be bedridden and lose economic productivity. Evidence shows that podoconiosis patients lose about 45% of their economic productivity (9). Despite its effect on an individual’s health and productivity and a country’s cumulative economy, the disease has received poor attention from both local and global communities (10).

The healthcare providers’ insights and skills equip them in the prevention and management of podoconiosis. However, different evidence showed that the healthcare providers had inadequate knowledge, a negative attitude, and poor practice toward podoconiosis management (11–13). Poor healthcare providers’ attitudes toward podoconiosis could result in stigma, compromise the quality of care, and overrun patients’ beliefs (14). Local evidence shows healthcare providers’ stigmatizing views and unfavorable attitudes toward podoconiosis management. For instance, a study conducted in northern Ethiopia found healthcare providers’ stigmatizing views toward patients with podoconiosis (12). Another study conducted in the Gamo Zone of southern Ethiopia revealed 44% unfavorable attitudes among healthcare providers (13). A study conducted by Yakob B. also revealed that less than one-half of healthcare providers had unfavorable attitudes (15). However, a study conducted in Rwanda revealed better (86.1% positive) attitudes toward podoconiosis management (11). Regarding podoconiosis management practices, the same study from Rwanda revealed that 6% of healthcare providers have ever managed podoconiosis cases (4). A study by Dellar et al. found healthcare providers’ poor practices in podoconiosis management (12). In the studies conducted in southern Ethiopia, 11.6 and 36% of healthcare providers ever managed patient with podoconiosis (13, 15).

The knowledge, attitude, and practice of podoconiosis management are affected by different sociodemographic and facility-related factors. The knowledge of podoconiosis management was significantly influenced by age, sex, type of profession, service year or experience of the provider, and the type of facility (private or public) (13, 15). Similar to factors influencing the knowledge of knowledge of podoconiosis management, the providers’ age, sex, knowledge of podoconiosis management, type of profession, ever managed podoconiosis, and type of health facility were found to be factors significantly affecting the attitudes toward podoconiosis management (13, 16, 17). The podoconiosis management practices were significantly affected by the sex of the provider and knowledge of podoconiosis (13).

Although the occurrence of podoconiosis in southwest Ethiopia, particularly in the Ilu Ababor and Buno Bedelle Zones, is higher, the knowledge, attitudes, and practices of podoconiosis have not been studied so far in the area. Hence, this study aimed at assessing the healthcare professionals’ knowledge, attitude, and practice of podoconiosis management and associated factors in public hospitals in Ilu Ababor and Buno Bedelle Zones, Southwest Ethiopia. The findings of this study could benefit the concerned bodies in endorsing and strengthening intervention strategies such as podoconiosis case management training for the healthcare professionals in the area.

## Materials and methods

### Study design, period, and area

A facility-based cross-sectional study was conducted in the public hospitals of Ilu Abba Bor and Buno Bedelle Zones. The study was executed in the period from December 1, 2022, to January 1, 2023. The two zones have an area of 15,135.33 square kilometers covered with 272,555 households. An estimated total of 2,271,609 people were living in the study area. Of these, about 636,986 and 634,623 men and women, respectively. The Ilu Ababor and Buno Bedelle Zones are the 20 Zones of the Oromia Regional State located in Southwest Ethiopia. The Ilu Ababor Zone has one referral [Mettu Karl Comprehensive Specialized Hospital (MKCSH)] and one district (Darimu Primary Hospital) hospital. These hospitals service communities of Southwest Ethiopia, including the Oromia Regional State, Gambella Regional State, Sheka, and Bench Maji Zones of Southwest Ethiopia Regional State. The MKCSH also serves as a pre- and in-services training center in the region. The Buno Bedlee Zone has three hospitals, including Bedelle General Hospital, Chora Primary Hospital, and Danbi Primary Hospital. A total of 451 healthcare professionals were working in the study area during the study period.

### Study participant selection and eligibility criteria

All randomly selected healthcare professionals who were working in the selected healthcare facilities and who lived for at least 6 months in the study area were included in this study. However, healthcare professionals who were not available (on annual, maternal, or sick leave) during the study period were excluded from this study.

### Sample size estimation and sampling procedure

A single population proportion formula $n=Z^{2}*\frac{P\left(1-p\right)}{d^{2}}$ , was used to estimate the sample size, assuming a 95% confidence level, a 5% margin of error, a 40% population proportion (P) taken from a study conducted in Gamo Zone (13), and a 10% non-response rate. A final sample size of 406 was used in this study.

Then the sample was proportionally allocated to each of the five public hospitals (MKCSH, Darimu Primary Hospital, Bedelle General Hospital, Chora Primary Hospital, and Didesa Primary Hospital) in the Ilu Ababor and Buno Bedelle Zones. The study participants were selected using a simple random sampling technique from each selected hospital (Table 1).

**Table 1:** Proportional allocation of the sample size to hospitals in Ilu Ababor and Buno Bedelle Zones, Southwest Ethiopia.

S/No.	Name of hospitals	Total number of health professionals in each hospital			Total	sample obtained from each hospitals			Total
		Doctors	Nurses	Midwives		Doctors	Nurses	Midwives	
1	MKCSH	30	132	27	189	27	119	25	171
2	BGH	19	69	19	107	17	62	17	96
3	DPH	8	42	12	62	7	38	11	56
4	DDPH	6	30	10	46	5	27	9	41
5	CPH	4	31	12	47	3	28	11	42
Total		67	304	80	451	59	274	73	406

BGH, Bedelle General Hospital; CPH, Chora Primary Hospital; DPH, Darimu Primary Hospital; DDPH, Didesa Primary Hospital; MKCSH, Mettu Karl Comprehensive Specialized Hospital.

### Study variables and outcome interest

Healthcare professionals’ knowledge, attitudes, and practices of podoconiosis management were the outcomes of this study. Sociodemographic factors (age, sex, salary, and marital status), profession-related factors (knowledge of podoconiosis, training on podoconiosis, type of profession, service year, level of education, job position, and having taken a course or topic about podoconiosis), and facility-related factors (level of the hospital and presence of management guidelines or manuals) were independent variables included in this study.

### Operational definitions

- Knowledge of podoconiosis management
  - Good knowledge: Participants who score 75% or more of the knowledge question.
  - Poor knowledge: Participants who score less than 75% of the knowledge questions (13).
- Attitude toward podoconiosis management
  - Favorable attitude: Participants who score 75% or more of attitude questions.
  - Unfavorable attitude: Participants who score below the 75% of attitude questions (13).
- Practice toward podoconiosis management
  - Practicing: Those participants who had ever treat podoconiosis patient.
  - Not practicing: Those participants who had never treated podoconiosis patient (13).

### Data collection instrument, procedure, and quality assurance

A structured, self-administered questionnaire was distributed to the selected participants. The questionnaire was adapted from a previous similar study conducted in southern Ethiopia (13). It contains five sections, including the participants’ sociodemographic information, participants’ knowledge of podoconiosis management among health professionals, participants’ attitudes toward podoconiosis management, participants’ podoconiosis management practices, and professional and facility-related information. Five BSc and two MSc-holder nurse professionals executed the data collection and supervision, respectively.

To ensure data quality, a pre-test was conducted among 5% of the sampled population. It was conducted 2 weeks before the actual data collection date at Gambella General Hospital. Then necessary amendments were made to the questionnaire based on the pre-test results. Data collectors and supervisors were given a one-day training regarding the study’s objectives, benefits, and participants’ rights. A daily check-up for data completeness and consistency was conducted during the data collection period.

### Statistical analysis

The collected data were coded and entered into Epi-data version 3.1. Then the data were exported to SPSS version 27.0 for analysis. Data cleaning and missed values were checked before analysis. The computed descriptive statistics were presented using text narrations, frequency tables, proportions, and graphs. The factors influencing knowledge, attitudes, and practice of podoconiosis management were tested using a binary logistic regression analysis. A variance inflation factor (VIF) analysis was conducted to assess multicollinearity. The bivariable analysis identified the candidate variables at a *p*-value of less than 0.25. The variables with a p-value of less than 0.05 in the multivariable analysis were considered statistically significant at a 95% confidence interval (CI). The adjusted odds ratio was used to show the magnitude and direction of statistical associations. The Hosmer-Lemeshow goodness-of-fit test was used to select the fitting model.

## Results

### Sociodemographic characteristics of participants

A total of 399 participants responded to the distributed (406 questionnaires), which gives a 98.2% response rate. About 218 (54.6%) of participants were between the ages of 25 and 35, with a mean (±SD) age of 31.73 (±6.31). More than two-thirds (271, 67.9%) of participants were nurses. About 153 (38.3%) of participants had 1–5 years of work experience. More than half (213, 53.4%) of the participants were earning a net salary of 5,000–10,000 Ethiopian Birr (ETB; Table 2).

**Table 2:** Sociodemographic characteristics of healthcare professionals in Ilu Ababor and Buno Bedelle Zones, Southwest Ethiopia (*n* = 399).

Variables	Frequency	Percentage
Sex		
Male	237	59.4
Female	162	40.6
Age in year (min = 22,max = 42,mean = 31.73, SD = 6.31)		
<25	91	22.8
25–35	218	54.6
>35	90	22.6
Year of experience (min = 1,max = 15, mean = 6.8, SD = 4.8)		
1–5	153	38.3
6–10 k	140	35.1
>10	106	26.6
Level of education		
Diploma	182	45.6
Degree	162	40.6
Masters and above	55	13.8
Type of profession		
Midwives	69	17.3
Nurses	271	67.9
Medical Doctors	59	14.8
Average monthly salary in ETB(Min = 4,609,Max = 12,000,Mean = 7,137)		
<50,000	169	42.4
5,000–10,000	213	53.4
>10,000	17	4.3
Took podoconiosis management training		
Yes	73	18.3
No	326	81.7
Having podoconiosis management guideline		
Yes	75	18.8
No	324	81.2
Took courses/tittle about podoconiosis		
Yes	16	4
No	383	96
Received periodic technical support from higher governing bodies		
Yes	64	16
No	335	84

ETB, Ethiopian Birr.

### Participants’ knowledge of podoconiosis management

About two-thirds (262, 65.7%) of participants had poor knowledge of podoconiosis management. About 150 (37.59%) of participants answered that podoconiosis is transmitted by contact with soil. About 160 (40%) stated avoiding walking barefoot in the cold as the means of podoconiosis prevention. The majority (364, 91.2%) of participants believed podoconiosis is a treatable disease (Table 3).

**Table 3:** Participants’ knowledge of podoconiosis management questions in Ilu Ababor and Buno Bedelle Zones, Southwest Ethiopia.

Questions	Responses	Frequency	Percentage
Have you ever heard of endemic/chronic elephantiasis or podoconiosis?	Yes	362	90.7
	No	37	9.3
Do any of these factors cause podoconiosis? (multiple options)			
Contact with affected patients		70	17.54
Mosquitoes		78	19.54
Contact with Soil		150	37.59
Spiritual cause/curse		11	2.75
Randomly occurring		22	5.5
Poverty		100	25.06
None of these apply		8	2
Which of these groups are most affected by podoconiosis? (multiple options)			
Adult men		60	15.03
Adult women		62	15.5
Children		58	14.5
Farmers		120	30
People who walk barefoot		78	19.5
People who do not wash their legs after contact with soil		54	13.5
None of these apply		20	5
Is podoconiosis preventable?	Yes	288	74.9
	No	100	25.1
If yes, what preventive measures do you know? (multiple options)			
Wash feet after contact with soil		105	26.3
Avoid contact with patients		68	17.04
Avoid walking on barefoot in the cold		160	40
Wear shoes		96	24.06
Avoid marriage with patients and their families^5^		15	3.7
Is podoconiosis treatable?	Yes	364	91.2
	No	35	8.8
What are signs and symptoms of podoconiosis? (multiple options)			
Reversible foot or leg swelling		50	12.5
Itching		70	17.5
Irreversible foot or leg swelling		56	14.03
burning sensations		69	17.2
lump growth/protrusions		80	20
Widening of foot		60	15.03
Loss of sensation on foot		50	12.5
Formation of skin folds		42	10.5
Knocking of big toes		25	6.25
Shallow skin folds		12	3
Plantar oedema		60	15.5
Total knowledge of podoconiosis management	Good knowledge	137	34.3%
	Poor knowledge	262	65.7%

### Participants’ attitudes toward podoconiosis management

More than half (213, 54.3%) of participants had unfavorable attitudes toward podoconiosis management. About 313 (78.4%) participants believed podoconiosis patients deserved love and support. The majority (346, 86.7%) of participants believed that people with podoconiosis deserved treatment and care (Table 4).

**Table 4:** Participants’ attitudes toward podoconiosis management questions in Ilu Ababor and Buno Bedelle Zones, Southwest Ethiopia.

Questions	Responses			
What comes to your mind when you see a patient requesting podoconiosis care?	Yes	Percentage	No	Percentage
The health provider is in danger of contracting podoconiosis	164	41.1	235	58.9
The patient is likely to be infective	170	42.6	229	57.4
The patient must have been sinful.	170	42.6	229	57.4
The patient is a responsible person.	219	54.9	135	33.8
Patients that contract podoconiosis have poor hygiene.	266	66.7	133	33.3
Patients with podoconiosis deserve love and support	313	78.4	86	21.6
If people knew that you treat podoconiosis patients, how would they treat you?	Yes	Percentage	No	Percentage
Isolate me	148	37.1	251	62.9
Appreciate your work	328	82.2	53	13.3
Suspect that you could have podoconiosis	219	54.9	180	45.1
Isolate my family members	25	6.3	374	93.7
Would you buy food or items from a shopkeeper with podoconiosis?	266	66.7	133	33.3
Would you feel happy if you were served food together with a podoconiosis patient?	304	76.2	95	23.8
Do you think you are at risk of acquiring podoconiosis for treating podoconiosis patients?	102	25.6	297	74.4
Do you agree or disagree with the following statements?	Agree	percentage	Disagree	Percentage
It is a person’s own fault if they developed podoconiosis	226	56.6	173	43.4
People with podoconiosis should be ashamed of themselves	146	36.6	253	63.4
People with podoconiosis can remain competitively productive members of society	385	96.5	14	3.5
People with podoconiosis should not feel guilt or shame	392	98.2	7	1.8
People with podoconiosis should be blamed for bringing the disease into the community	147	36.8	252	63.2
Our society does not provide enough help to people with podoconiosis	351	88	48	12
People who say they are podoconiosis patients are brave and strong	259	64.9	140	35.1
People with podoconiosis are a threat to their own health and their families health	214	53.6	185	46.4
People with podoconiosis deserve sympathy	357	89.5	42	10.5
People with podoconiosis deserve treatment and care	346	86.7	53	13.3
The family of the person with podoconiosis is also to blame	133	33.3	266	66.7
The family of the person with podoconiosis is cursed and should be avoided & isolated	143	35.8	256	64.2
People with podoconiosis are sinners/wrongdoers	145	36.3	254	63.7
People with podoconiosis should be legally separated from others to protect the public health	150	37.6	249	62.4
Total attitudes toward podoconiosis management	Favorable		186	46.6%
	Unfavorable		213	53.4%

### Participants’ podoconiosis management practices

More than three-fourths (308, 77.2%) of participants did not ever practice podoconiosis. Of those who ever treated podoconiosis patients, only 62 (15.5%) perceived they were confident in delivering services to podoconiosis patients. More than two-thirds (270, 67.6%) of participants mentioned the unavailability of drugs and supplies as the challenges they faced while treating podoconiosis patients (Table 5).

**Table 5:** Participants’ podoconiosis management practices in Ilu Ababor and Buno Bedelle Zones, Southwest Ethiopia.

Variables		Response		Percentage
Have you ever treated patient with podoconiosis		Yes	91	22.8
		No	308	77.2
Are you confident in your ability to deliver services to podoconiosis patient?		Yes	62	15.5
		No	29	7.26
If no, what prevents you from delivering good services to podoconiosis patients? (check all that applies)				
I do not have skills and/or knowledge		149		37.3
I fear contracting podoconiosis		35		8.7
I lack essential materials and supplies		230		57.6
I do not want to treat podoconiosis patients		10		2.5
What challenges do you encounter while treating podoconiosis patients? (check all that apply)				
Patients do not accept care and treatment		16		4
Drugs and supplies are not available		270		67.6
Unpleasant smell, discomfort at work		50		12.5
Number of patients is low		10		2.5
Do you have the necessary materials, supplies and equipment to deliver good services to podoconiosis patients?		Yes	35	8.7
		No	56	14
This health facility provides everything I need to deliver a good service to podoconiosis patients effectively		Yes	31	7.7
		No	60	15.03
How would you manage an acute attack of podoconiosis? (Check all that apply)				
Prescribed antibiotics		50		12.53
Surgical treatment		12		3
Prescribed Diethyl carbamazine (DEC)		45		11.27
Referral to other treatment facility		30		7.5
How would you manage a chronic attack of podoconiosis?(Check all that apply)				
ointments/soaps for topical care		60		15.03
Surgical treatment		15		37.6
Referred to other treatment facility		40		10
On average how many podoconiosis patients do you treat per month?	1–2 times	40		10
	3-4times	35		8.7
	>5	16		4
Total practice of podoconiosis management	Practiced	91		22.8%
	Not practiced	308		77.2%

### Factors associated with participants’ knowledge of podoconiosis management

Participants with more than 10 years of work experience were more than twofold [adjusted odds ratio (AOR): 2.2; 95% CI: 1.13–4.27] more knowledgeable than those with 1–5 years of work experience. Participants who had masters and above were nearly five [AOR: 4.8; 95%CI: 2.12–10.97] times more likely to be knowledgeable than those who had diplomas. Participants with medical doctor qualifications were more than fivefold [AOR: 5.3; 95%CI: 2.10–13.82] more likely to be knowledgeable than midwives. Participants who took podoconiosis training were about twofold [AOR: 2.1; 95%CI: 1.1–4.1] more likely to be knowledgeable compared to their counterparts. Participants who took podoconiosis management courses or titles in their educational curricula had a more than threefold [AOR: 3.6; 95%CI: 1.09–12.36] likelihood of having good knowledge of podoconiosis management. Moreover, participants with favorable attitudes toward podoconiosis management had a sevenfold [AOR: 7.1; 95%CI: 4.22–12.19] higher probability of having good knowledge compared to those with unfavorable attitudes (Table 6).

**Table 6:** Factors associated with participants’ knowledge of podoconiosis management in Ilu Ababor and Buno Bedelle Zones, Southwest Ethiopia.

Variables	Categories	Knowledge of podoconiosis management		COR (95%C.I)	AOR (95%C.I)
		Poor knowledge	Good knowledge		
Sex	Female	107	55	1	
	Male	155	82	1.029 (0.67–1.56)	
Age in year	<25	67	24	1	
	25–35	157	61	1.085 (0.62–1.88)	
	>35	38	52	1.82 (0.9–2.14)	
Year of experience	1–5	119	34	1	1
	6–10	85	55	2.265 (1.36–3.77)**	1.8 (0.96–3.38)
	>10	58	48	2.89 (1.68–4.9)**	2.2 (1.13–4.27)*
Level of education	Diploma	136	46	1	1
	Degree	109	53	1.43 (0.9–2.3)	1.1 (0.67–2.11)
	Masters	17	38	6.6 (3.4–128)*	4.8 (2.12–10.97)**
Profession	Midwives	56	13	1	1
	Nurses	184	87	2.03 (1.05–3.9)*	1.9 (0.9–4.01)
	Medical Doctors	22	37	7.24 (3.2–16.1)**	5.3 (2.10–13.82)**
monthly salary	<50,000	108	61	1	
	5,000–10,000	144	69	0.84 (0.55–1.29)	
	>10,000	10	7	1.23 (0.44–3.42)	
Took training	No	235	91	1	1
	Yes	27	46	4.4 (2.5–7.4)**	2.1 (1.1–4.1)*
Having guideline	No	232	92	1	
	Yes	30	45	3.7 (2.24–6.37)**	
Taking course	No	255	128	1	1
	Yes	7	9	2.5 (0.9–7.03)	3.6 (1.09–12.36)**
Received technical support	No	229	106	1	
	Yes	33	31	2.02 (1.18–3.48)	
Attitude toward podoconiosis management.	Non favorable	183	30	1	1
	Favorable	79	107	8.2 (5.09–13.39)***	7.1 (4.22–12.19)**

COR, Crude odds ratio; AOR, Adjusted odds ratio.

### Factors associated with participants’ attitudes toward podoconiosis management

In this study, participants above 35 were 1.5 [AOR: 1.5; 95%CI: 1.13–3.52] times more likely to have favorable attitudes toward podoconiosis management than those under 25. The probability of having favorable attitudes was about six fold [AOR: 5.9; 95%CI: 1.1–13] higher among participants who had podoconiosis training than their counterparts. Participants who received technical support had nearly two [AOR: 1.8; 95%CI: 1.1–3.2] times higher likelihood of unfavorable attitudes than those who had not received technical support. In addition, participants with good knowledge of podoconiosis management had a nearly eightfold [AOR: 7.7; 95%CI: 4.6–12] higher likelihood of having favorable attitudes (Table 7).

**Table 7:** Factors associated with participants’ attitudes toward podoconiosis management in Ilu Ababor and Buno Bedelle Zones, Southwest Ethiopia.

Variables	Categories	Attitudes toward podoconiosis management		COR (95%C.I)	AOR (95%C.I)
		Non favorable	Favorable		
Sex	Female	82	80	1	
	Male	131	106	1.2 (0.8–1.8)	
Age in year	<25	56	35	1	
	25–35	122	96	1.2 (0.7–2.07)	
	>35	35	55	2.5 (1.3–4.5)**	1.5 (1.13–3.52)**
Year of experience	1–5	95	58	1	
	6–10	70	70	1.6 (1.02–2.6)**	
	>10	48	58	1.9 (1.19–3.27)**	
Level of education	Diploma	98	84	1	
	Degree	96	66	0.8 (0.5–1.20)	
	Masters	19	36	2.2 (1.18–4.14)**	
Current qualification	Midwifery	39	30	1	
	Nurses	154	117	0.99 (0.0.57–1.68)	
	Medical Doc	20	39	2.5 (1.2–5.2)**	
monthly salary	<50,000	80	89	1	
	5,000–10,000	126	87	0.6(0.0.41–0.93)	
	>10,000	7	10	1.28 (0.4–3.5)	
Taking podoconiosis Training	No	118	138	1	1
	Yes	25	48	2.6 (1.53–4.44)**	5.9 (1.1–13)**
Having guideline	No	183	141	1	1
	Yes	30	45	1.9 (1.16–3.24)**	
Having course	No	206	177	1	
	Yes	7	9	1.4 (0.56–4.1)	
Received technical support	No	189	146	1	1
	Yes	24	40	2.1 (1.24–3.74)**	1.8 (1.1–3.2)**
Level of Knowledge	Poor	183	79	1	1
	Good	30	107	8.2 (5.09–13.39)***	7.7 (4.6–12)**

COR, Crude odds ratio; AOR, Adjusted odds ratio.

### Factors associated with participants’ podoconiosis management practices

Participants above 35 were three [AOR: 3.05; 95%CI: 1.35–6.90] times more likely to practice podoconiosis management than those under 25. Participants who had master’s degrees and above were more than two [AOR: 2.4; 95%CI: 1.07–5.48] times more likely to practice podoconiosis management than those who had diplomas. Participants who took podoconiosis training were more than three [AOR: 3.6; 95%CI: 1.81–7.45] times more likely to practice podoconiosis management than their counterparts. Participants who took podoconiosis courses or titles in educational curricula were about threefold [AOR: 3.1; 95%CI: 1.2–10.61] more likely to practice podoconiosis management than those who had not taken the course in their current education. In addition, participants with good knowledge of podoconiosis were twofold [AOR: 2.3; 95%CI: 1.27–4.12] more likely to practice podoconiosis management than their counterparts (Table 8).

**Table 8:** Factors associated with participants’ podoconiosis management practices in Ilu Ababor and Buno Bedelle Zones, Southwest Ethiopia.

Variables	Categories	Podocoinosis management practice		COR (95%C.I)	AOR (95%C.I)
		Not practicing	Practicing		
Sex	Female	120	42	1	
	Male	188	49	1.3 (0.8–2.15)	
Age in year	<25	78	13	1	1
	25–35	187	31	1.1 (0.5–2)	1.1 (0.5–2.001)
	>35	43	47	6.5 (3.19–13.44)**	3.05 (1.35–6.90)**
Year of experience	1–5	122	31	1	
	6–10	111	29	1.02 (0.58–1.81)	
	>10	75	31	1.62 (0.91–2.89)	
Level of education	Diploma	153	29	1	1
	Degree	132	30	1.19 (0.68–2.10)	1.1 (0.5–1.59)
	Masters	23	31	7.34 (3.76–14.29)**	2.4 (1.07–5.48)**
Current qualification	Midwifery	57	12	1	
	Nurses	208	63	1.43 (0.72–2.84)	
	Medical Doctors	43	16	1.76 (0.75–4.12)	
monthly salary	<50,000	129	40	1	
	5,000–10,000	164	49	0.96 (0.59–1.55)	
	>10,000	12	5	0.43 (0.09–1.96)	
Taking training	No	276	50	1	1
	Yes	32	41	7.07 (4.07–12.28)**	3.6 (1.81–7.45)**
Having guideline	No	272	52	1	
	Yes	36	39	5.6 (3.29–9.73)**	1
Taking course	No	298	85	1	
	Yes	10	6	2.1 (0.7–5.9)**	3.1 (1.2–10.61)**
Received technical support	No	263	72	1	
	Yes	45	19	1.54 (0.85–2.80)	
Level of Knowledge	Poor	277	35	1	1
	Good	81	56	4.48 (2.74–7.33)	2.3 (1.27–4.12)**
Level of attitude	Non favorable	180	33	1	
	Favorable	128	58	2.47 (1.52–4.009)**	

COR, Crude odds ratio; AOR, Adjusted odds ratio.

## Discussion

The present study aimed to assess the participant’s knowledge, attitude, and practice toward podoconiosis management and associated factors in the Ilu Ababor and Buno Bedelle Zones of southwest Ethiopia. About 65.7% of healthcare professionals had poor knowledge of podoconiosis. The current study reveals a higher level of poor knowledge about podoconiosis than a Rwandan study (11). This variation could be related to the differences in health education curricula, training accessibility, and technical support for healthcare professionals in the two countries. However, the current proportion of poor knowledge of podoconiosis is congruent with the previous findings from the Gamo (13) and Wolaita (15) Zones. It might be associated with a lack of pre- and in-service training about podoconiosis. Therefore, pre- and in-service training should be given to improve health professionals’ podoconiosis management knowledge.

In the current study, years of experience were significantly associated with knowledge of podoconiosis management. The odds of podoconiosis management knowledge were 2.2 times higher among participants with more than 10 years of experience than those with 1 to 5 years of experience. This was a plausible finding because knowledge of a certain disease condition increases as years of experience increase because of repeated exposure to different case management in clinical settings. This finding was supported by a study conducted in Gamo Zone (13). However, this association was not supported by a study finding from the Wolaita Zone, southern Ethiopia (15). Level of education was another factor that showed a significant association with knowledge of podoconiosis management in this study. Participants who studied for master’s degrees had 4.8 times higher odds of podoconiosis management knowledge than those with diploma certificates. This association was a novel and has not been supported by earlier literature. The finding was based on the fact that the knowledge level increases with education. It could also be due to the variations in the contents of the curriculum at different levels of degrees. In a previous study, public health officers, BSc nurses, and diploma nurses had a higher probability of having good podoconiosis management knowledge than other healthcare professions (13). However, medical doctors had more than fivefold odds of good knowledge of podoconiosis management in this study. This could be related to the curriculum variations among the healthcare professional educations in the country. Participants who took training had two times the odds of podoconiosis management knowledge than their counterparts. The training was not significantly associated with knowledge of podoconiosis management in a previous study (13). Pre- and in-service training are crucial in boosting the knowledge and skills of healthcare professionals in case management. Healthcare professionals who learned podoconiosis in course materials had a 3.6 times higher probability of having good knowledge of podoconiosis management. This could be due to recalling the previous knowledge of podoconiosis. Since this was a novel attribute of this study, no previous evidence supported it. Participants who had favorable attitudes toward podoconiosis management had sevenfold higher odds of podoconiosis management knowledge compared to their counterparts. This significant association was also found in a study conducted in the Gamo Zone (13).

The study identified healthcare professionals’ attitudes toward podoconiosis management. Accordingly, more than half (53.4%) of the healthcare professionals had an unfavorable attitude. This finding is comparable to a finding from northern Ethiopia, where healthcare provides stigmatizing views toward podoconiosis patients (12). However, it is higher compared to a study by Yakob B. et al., where about 48% of healthcare professionals had unfavorable attitudes (15). The current proportion is higher than that of the proportion found in the Gamo Zone, where around 44% of healthcare professionals had an unfavorable attitude (13). Moreover, the healthcare providers’ unfavorable attitude in the area was more than threefold higher than in Rwanda’s finding, where only 13.9% had an unfavorable attitude (11). This variation could be associated with a lack of podoconiosis management training for the professionals in the study setting.

Healthcare professionals who were under 25 years old had 1.5 times higher odds of a favorable attitude toward podoconiosis management compared to those who were 35 years and older. This is a brand-new discovery that has not been corroborated by earlier literature. The likelihood of having a positive attitude was around six times higher among healthcare professionals with training than their counterparts. Previous literature did not provide evidence for this. The training improves the attitudes toward the phenomenon. Receiving technical support was also demonstrated to have a strong correlation with a favorable attitude toward podoconiosis management. As a result, compared to healthcare workers who did not receive technical support, those who did had roughly two-fold higher odds of having favorable attitudes toward podoconiosis management. In addition, the podoconiosis management knowledge of healthcare professionals was identified as a significant factor affecting the professionals’ attitudes. Healthcare professionals who had good knowledge of podoconiosis management had nearly eight-fold higher likelihood of having a favorable attitude. This finding asserts that a higher level of knowledge about a certain phenomenon increases the attitude toward the condition.

In this study, more than three-fourths (77.20%) of healthcare professionals were not practicing podoconiosis management. The practice proportion found in this study is higher than the previous finding from the Wolaita Zone, where 64% of healthcare providers did not practice podoconiosis management (15). However, the current proportion is lower than a finding from Rwanda, where only 94% of the healthcare professionals did not practice podoconiosis management (11). This difference might be related to the variations in the distribution of podoconiosis cases in the two countries, health service coverage, and availability of in-service and pre-service training.

Participants whose age was 35 years and above were three times more likely to practice podoconiosis management compared to those whose age was less than 25 years. This finding agrees with the findings of the Gamo and Wolaita Zones (13, 15). This could be due to healthcare professionals’ increased exposure to podoconiosis patients for a long period of time. Educational status was found to be another factor affecting professionals’ podoconiosis management practice. Participants who held master’s degrees had a roughly twofold higher chance of practicing podoconiosis management than those who held diplomas. This association is similar to the truth that a higher level of education leads to higher practice competence. Participants who took training had about 3.6 times higher probability of practicing podoconiosis management than those who did not take training. This finding was supported by a study conducted by Churko et al. (13). Participants who took podoconiosis courses in their curriculum had threefold higher odds of practicing podoconiosis management than their counterparts. Moreover, participants with good knowledge of podoconiosis management had a twofold higher likelihood of practicing podoconiosis management than those with poor knowledge. This finding is consistent with a study by B. Yakob et al. (15).

### Limitations of the study

The distribution of self-administered questionnaires could be subject to response bias from each respondent. Since the study utilized a cross-sectional design, it could not assert a temporal relationship between the outcome and explanatory variables. Moreover, these qualitative findings could not explore possible sociocultural barriers to podoconiosis management.

## Conclusion

Podoconiosis is a neglected disease both in a local and global context. Although podoconiosis cases are prevalent in the study area, the healthcare professionals’ knowledge, attitudes, and practice of podoconiosis management were found to be poor. Given that building the capacity of healthcare professionals is crucial to the prevention and treatment of podoconiosis, the concerned bodies, including the federal and regional health bureaus and other non-governmental organizations, should endorse podoconiosis training opportunities and provide podoconiosis management guidelines to the healthcare professionals. The Ministry of Education, in collaboration with the Ministry of Health, shall also include podoconiosis in the educational curricula. At the individual level, healthcare professionals should struggle to improve their knowledge and skills in managing podoconiosis patients. Moreover, researchers in the field need to undertake follow-up studies and explore further contributors to healthcare professionals’ inadequacies in podoconiosis management.

## Data Availability

The original contributions presented in the study are included in the article/Supplementary material, further inquiries can be directed to the corresponding author.
