# Supplementary material for: Healthcare professionals’ knowledge, attitudes, and practice of podoconiosis management and associated factors in public hospitals in Ilu Ababor and Buno Bedelle zones, Southwest Ethiopia: a cross-sectional study
Source: Front Public Health. 2025 Feb 24;13:1454979. doi: 10.3389/fpubh.2025.1454979 (PMC11891203; doi:10.3389/fpubh.2025.1454979)
Supplement: Supplementary file 1 [file Table_1.docx]

**Information sheet**

**Introduction**: Greeting! I am working as a data collector in a research entitled “***Healthcare professionals’ knowledge, attitudes, and practice of podoconiosis management and associated factors in public hospitals in Ilu Ababor and Buno Bedelle Zones, Southwest Ethiopia: A cross-sectional study***”. You are selected as a participant in this study. Thus before giving your consent or permission for participation, you need to know all the necessary information related to the study.

**Objective:** The general objective of this study is toassess knowledge, attitude and practice toward podoconosis management and its associated factors among health professionals working in Ababor and Buno Bedelle Zones, Southwest Ethiopia, 2022.

**Name of the organization**: Mattu University, college of health science.

**Participants:** Selected health professional working hospitals of Illubabor and Buno Bedelle Zones.

**Confidentiality**: All information you give will not be accessible to any third party and kept confidential. . You are not asked to write your name on the questionnaire sheet so that you will not be identified

**Risks***:* The procedure does not bear any physical or psychological trauma on you. You are not forced to respond to the information you do not know. However, by participating in this research project, you may feel that it is time consuming, wasting about 30 minutes. We hope you will participate in the study for the sake of the benefit of the research result

**Benefits**: For your participation in the study, no payment will grant. However, participating in the study and giving your information to questions asked will have great input in efforts to identify level of KAP toward podoconosis management and the result of the study will help in improving care of patients with podoconosis.

**Right to refuse or withdraw: -** Your participation is voluntary and you are not obligated to answer any question you do not wish to answer. If you feel discomfort with the question, it is your right to drop it any time you want. If you have questions regarding this study or would like to be informed of the results after its completion, please feel free to contact the principal investigator.

**Person to contact:**

If you have any question to ask, please contact

1. SanbatoTamiru; , Phone No:0975251660, E-mail: tsanbato@yahoo.com
2. Bonsa Amsalu, phone No:0917442400, E-mail bonsa43@gmail.com

**Do you want to participate?**

_________ Yes, I want to participate in the study (Please go to the next page).

_________ No, I do not participate in the study (Thank you very much!)
