# Supplementary material for: Healthcare professionals’ knowledge, attitudes, and practice of podoconiosis management and associated factors in public hospitals in Ilu Ababor and Buno Bedelle zones, Southwest Ethiopia: a cross-sectional study
Source: Front Public Health. 2025 Feb 24;13:1454979. doi: 10.3389/fpubh.2025.1454979 (PMC11891203; doi:10.3389/fpubh.2025.1454979)
Supplement: Supplementary file 2 [file Table_2.docx]

**Questionnaire**

|  | **Section 1: Demographic information and working experience** | | |
| --- | --- | --- | --- |
| **Respondents** | **No.** | **QUESTIONS** | **CODING CLASSIFICATION** |
|  | 101 | What is your age? | ____________ yrs |
|  | 102 | What is your sex?  (Please check one) | 🞎 Male^1^ 🞎 Female^2^ |
|  | 103 | How many years have you worked in this profession? | ______________yrs🞎Refuse to answer |
|  | 104 | What is your highest level of education?  (Please check one) | 🞎Bachelor degree^3^🞎Master degree^4^ |
|  |  |  | Other(specify)^5^_______________________ |
|  | 105 | What is your current technical qualification?  (Please check one) | 🞎 General practitioner^1^ 🞎Specialist doctor^2^  🞎 HO🞎Nurse |
|  |  |  | Other (specify)^7^________________________ |
|  | 106 | How much is your monthly salary | ______________BR |

**Section 2**: **Knowledge Questions**

| **No.** | **QUESTIONS** | | | **CODING CLASSIFICATION** |
| --- | --- | --- | --- | --- |
| 201 | Have you ever heard of endemic/chronic elephantiasis (podoconiosis)? (Please check one) | | | 🞎 Yes^1^ 🞎 No^2^ 🞎 I don’t know^3^ |
| 202 | 1. Please describe the causes of podoconiosis. | | | _____________________________  🞎 I don’t know |
|  | 1. Do any of these factors cause podoconiosis? (check all that apply)   🞎 Heredity^1^🞎 Contact with affected patients^2^ 🞎 Mosquitoes^3^  🞎 Soil^4^🞎 Spiritual cause/curse^5^ 🞎 Randomly occurring^6^  🞎 Poverty^7^🞎 None of these apply^9^🞎 I don’t know^9^ | | | |
| 203 | 1. Who is at highest risk of podoconiosis? | | _______________________________  🞎 I don’t know | |
|  | 1. Which of these groups are most affected by podoconiosis?   (Check all that apply)  🞎Adult men^1^🞎 Adult women^2^🞎 Children^3^  🞎Farmers^4^ 🞎 People who walk barefoot^5^  🞎 People who don’t wash their legs after contact with soil^5^  🞎 None of these apply^8^🞎 I don’t know^8^ | | | |
| 204 | 1. Is podoconiosis preventable? | | 🞎 Yes^1^ 🞎 No^2^ 🞎 I don’t know^3^ | |
|  | 1. If yes, what preventive measures do you know? (check all that apply)   🞎 Wash feet after contact with soil^1^🞎 Avoid contact with patients^2^  🞎 Avoid walking on barefoot in the cold^3^🞎 Wear shoes^4^  🞎 Avoid marriage with patients and their families^5^🞎 I don`t know^6^ | | | |
|  | Other (specify)^7^_____________ | | | |
| 205 | Is podoconiosis treatable? | 🞎 Yes^1^ 🞎 No^2^ 🞎 I don’t know^3^ | | |
| 206 | What are signs and symptoms of podoconiosis? (check all that apply)  🞎Reversible foot or leg swelling^1^🞎 Itching^2^  🞎 Irreversible foot or leg swelling^3^ 🞎 burning sensations^4^  🞎 lump growth/protrusions^5^🞎 Widening of foot^6^  🞎Loss of sensation on foot^7^🞎 Formation of skin folds^8^  🞎 Knocking of big toes^9^ 🞎 Shallow skin folds^10^  🞎 Plantar oedema^11^🞎 Large second toes^12^  🞎 I don’t know^13^ | | | |
|  | Other (specify)^14^__________________________ | | | |

**Section 3: Attitude Questions**

| **No.** | **QUESTIONS** | **CODING CLASSIFICATION** |
| --- | --- | --- |
| 301 | What comes to your mind when you see a patient requesting podoconiosis care? |  |
|  | 1. The health provider is in danger of contracting podoconiosis | 🞎 Yes^1^ 🞎 No^2^ 🞎 I don’t know^3^ |
|  | 1. The patient is likely to be infective | 🞎 Yes^1^ 🞎 No^2^ 🞎 I don’t know^3^ |
|  | 1. The patient must have been sinful. | 🞎 Yes^1^ 🞎 No^2^ 🞎 I don’t know^3^ |
|  | 1. The patient is a responsible person. | 🞎 Yes^1^ 🞎 No^2^ 🞎 I don’t know^3^ |
|  | 1. Patients that contract podoconiosis have poor hygiene. | 🞎 Yes^1^ 🞎 No^2^ 🞎 I don’t know^3^ |
|  | 1. Patients with podoconiosis deserve love and support | 🞎 Yes^1^ 🞎 No^2^ 🞎 I don’t know^3^ |
|  | 1. Other (specify)__________________________________________ | |
| 302 | If people knew that you treat podoconiosis patients, how would they treat you? |  |
|  | 1. Isolate me | 🞎 Yes^1^ 🞎 No^2^ 🞎 I don’t know^3^ |
|  | 1. Appreciate your work | 🞎 Yes^1^ 🞎 No^2^ 🞎 I don’t know^3^ |
|  | 1. Suspect that you could have podoconiosis | 🞎 Yes^1^ 🞎 No^2^ 🞎 I don’t know^3^ |
|  | 1. Isolate my family members | 🞎 Yes^1^ 🞎 No^2^ 🞎 I don’t know^3^ |
|  | 1. Other (specify)____________________________________________ | |
| 303 | Would you buy food or items from a shopkeeper with podoconiosis? | 🞎 Yes^1^ 🞎 No^2^ 🞎 I don’t know^3^ |
| 304 | Would you feel happy if you were served food together with a podoconiosis patient? | 🞎 Yes^1^ 🞎 No^2^ 🞎 I don’t know^3^ |
| 305 | 1. Do you think you at risk of acquiring podoconiosis? | 🞎 Yes^1^ 🞎 No^2^ 🞎 I don’t know^3^ |
|  | 1. If yes, why? | ____________________________ |
|  | 1. If no, why? | ____________________________ |
| 306 | Do you agree or disagree with the following statements? |  |
|  | 1. It is a person`s own fault if they developed podoconiosis | 🞎 Agree^1^ 🞎 Disagree^2^ 🞎 I don`t know^3^ |
|  | 1. People with podoconiosis should be ashamed of themselves | 🞎 Agree^1^ 🞎 Disagree^2^ 🞎 I don`t know^3^ |
|  | 1. People with podoconiosis can remain competitively productive members of society | 🞎 Agree^1^ 🞎 Disagree^2^ 🞎 I don`t know^3^ |
|  | 1. People with podoconiosis should not feel guilt or shame | 🞎 Agree^1^ 🞎 Disagree^2^ 🞎 I don`t know^3^ |
|  | 1. People with podoconiosis should be blamed for bringing the disease into the community | 🞎 Agree^1^ 🞎 Disagree^2^ 🞎 I don`t know^3^ |
|  | 1. Our society does not provide enough help to people with podoconiosis | 🞎 Agree^1^ 🞎 Disagree^2^ 🞎 I don`t know^3^ |
|  | 1. People who say they are podoconiosis patients are brave and strong | 🞎 Agree^1^ 🞎 Disagree^2^ 🞎 I don`t know^3^ |
|  | 1. People with podoconiosis are a threat to their own health and their families health | 🞎 Agree^1^ 🞎 Disagree^2^ 🞎 I don`t know^3^ |
|  | 1. People with podoconiosis deserve sympathy | 🞎 Agree^1^ 🞎 Disagree^2^ 🞎 I don`t know^3^ |
|  | 1. People with podoconiosis deserve treatment and care | 🞎 Agree^1^ 🞎 Disagree^2^ 🞎 I don`t know^3^ |
|  | 1. The family of the person with podoconiosis is also to blame | 🞎 Agree^1^ 🞎 Disagree^2^ 🞎 I don`t know^3^ |
|  | 1. The family of the person with podoconiosis is cursed and should be avoided & isolated | 🞎 Agree^1^ 🞎 Disagree^2^ 🞎 I don`t know^3^ |
|  | 1. People with podoconiosis are sinners/wrongdoers | 🞎 Agree^1^ 🞎 Disagree^2^ 🞎 I don`t know^3^ |
|  | 1. People with podoconiosis should be legally separated from others to protect the public health | 🞎 Agree^1^ 🞎 Disagree^2^ 🞎 I don`t know^3^ |

**Section 4: Practice questions**

| **No.** | **QUESTIONS** | | **CODING CLASSIFICATION** | | |
| --- | --- | --- | --- | --- | --- |
| **401** | Have you ever treated patient with podoconiosis | | 🞎 Yes→402 🞎 No | | |
| 402 | 1. Are you confident in your ability to deliver services to podoconiosis patient? | | 🞎 Yes^1^ 🞎 No^2^ 🞎 I don’t know^3^ | | |
|  | 1. If no, what prevents you from delivering good services to podoconiosis patients? (check all that applies)   🞎I don’t have skills and/or knowledge^1^  🞎 I fear contracting podoconiosis^2^  🞎 I lack essential materials and supplies^3^  🞎 I don’t want to treat podoconiosis patients^4^  🞎I fear stigma and discrimination for treating patients^5^ | | | | |
|  | Other (specify)^6^_________________________________________ | | | | |
| 403 | Have you received sufficient training to be able to perform your job well regarding podoconiosis management and prevention? | 🞎 Yes^1^ 🞎 No^2^ 🞎 Don’t know^3^ | | | |
| 404 | Do you have the necessary materials, supplies and equipment to deliver good services to podoconiosis patients? | 🞎 Yes^1^ 🞎 No^2^ 🞎 Don’t know^3^ | | | |
| 405 | What challenges do you encounter while treating podoconiosis patients?  (check all that apply)  🞎Patients do not accept care and treatment^1^  🞎Drugs and supplies are not available^2^  🞎Unpleasant smell, discomfort at work^3^  🞎Discomfort at work^4^  🞎Number of patients is low^5^ | | | | |
|  | Other( specify)^6^____________________________________________ | | | | |
| 406 | This health facility provides everything I need to deliver a good service to podoconiosis patients effectively | | | 🞎 Yes^1^ 🞎 No^2^ 🞎 Don’t know^3^ | |
| 407 | If no to 403, what is lacking? | | | ……….. | |
| 408 | 1. How would you manage an acute attack of podoconiosis?   (Check all that apply)  🞎 Prescribed antibiotics^1^ 🞎 Surgical treatment^2^  🞎 Prescribed Diethyl carbamazine (DEC)^3^🞎 Lab investigations^4^  🞎 Referral to other treatment facility^5^ 🞎 I don’t know^6^ | | | | |
|  |  |  |  |  |  |
|  | Other (specify)^7^___________________________________________ | | | | |
|  | 1. How would you manage a chronic attack of podoconiosis?   🞎 ointments/soaps for topical care^1^🞎 Surgical treatment^2^  🞎 Referred to other treatment facility^3^ 🞎I don’t know^4^ | | | | |
|  | Other (specify)^5^___________________________________________ | | | | |
| 409 | On average how many patients with podoconiosis do you treat per month? | | | | _______________________ |
| 410 | Is there anything else you would like to tell us about your experiences with podoconiosis? | | | | _________________________ |

**Section III: - Other factors associated with KAP of podoconosis management**

| 501 | Have you ever received any type of training regarding podoconisismangment? |
| --- | --- |
|  | 1. Yes B. No |
| 502 | Have you taken course/ topic on podoconosis management during your education before your current graduation? |
|  | 1. Yes B. No |
| 503 | Do you have any type of guideline/manual that you refer and help you to providea care for patient with podoconosis? |
|  | 1. Yes B. No |
| 504 | Have you ever received any technical support/supervision regarding podoconosis management from higher official? |
|  | 1. Yes B. No |

Thank you for participating in this study!

Collected by

Name of the data collector_________________________

Signature___________________________________

Date ______________________________
